# Supplementary material for: From sequence to enzyme mechanism using multi-label machine learning
Source: BMC Bioinformatics. 2014 May 19;15:150. doi: 10.1186/1471-2105-15-150 (PMC4229970; doi:10.1186/1471-2105-15-150)
Supplement: Additional file 2 — Java code of ml2db. Additional file ml2db_code.tar.gz contains the Java source code to run the multi-label machine learning experiments and save the results to database. The code’s Javadoc is included. [file 1471-2105-15-150-S2.zip › additional file 2/ml2db/utils/doc/index-files/index-12.html]

L-Index


JavaScript is disabled on your browser.


- Overview
- Package
- Class
- Use
- Tree
- Deprecated
- Index
- Help

- Prev Letter
- Next Letter

- Frames
- No Frames

- All Classes

A B C D E F G H I J K L M N O P Q R S T U V W X Y 


## L

leftChopString(String, int) - Static method in class uk.ac.standrews.utils.main.stringutils.StringUtils
:   Shortens the given string to the number of characters specified by
    chopping off characters on the left, if needed.

list() - Static method in class uk.ac.standrews.utils.test.ListUtilsTest


listFromVector(Vector<String>) - Static method in class uk.ac.standrews.utils.main.collectionutils.VectorUtils


ListUtils - Class in uk.ac.standrews.utils.main.collectionutils
:   Utilities to manipulate lists

ListUtils() - Constructor for class uk.ac.standrews.utils.main.collectionutils.ListUtils


ListUtilsTest - Class in uk.ac.standrews.utils.test
:   Class

ListUtilsTest() - Constructor for class uk.ac.standrews.utils.test.ListUtilsTest


log(Object) - Static method in class uk.ac.standrews.utils.main.LogUtils


log(Object, String) - Static method in class uk.ac.standrews.utils.main.LogUtils


log(String) - Static method in class uk.ac.standrews.utils.main.LogUtils


logAndPrint(File, String) - Static method in class uk.ac.standrews.utils.main.LogUtils


logAndPrint(String, String) - Static method in class uk.ac.standrews.utils.main.LogUtils


logAndPrint(File, String, boolean) - Static method in class uk.ac.standrews.utils.main.LogUtils
:   Logs some text to file AND prints it to screen.

logAndPrintNoTimestamp(File, String) - Static method in class uk.ac.standrews.utils.main.LogUtils


LoggerCreator - Class in uk.ac.standrews.utils.main


logMilliseconds(String) - Static method in class uk.ac.standrews.utils.main.LogUtils


LogUtils - Class in uk.ac.standrews.utils.main
:   Utilities to log error messages

LogUtils() - Constructor for class uk.ac.standrews.utils.main.LogUtils


LONG\_TEXT\_SQL\_DATATYPE - Static variable in class uk.ac.standrews.utils.main.database.DbUtils


lxdflt - Static variable in class edu.cornell.lassp.houle.RngPack.Ranlux
:   Default luxury level: `lxdflt=3`

A B C D E F G H I J K L M N O P Q R S T U V W X Y

- Overview
- Package
- Class
- Use
- Tree
- Deprecated
- Index
- Help

- Prev Letter
- Next Letter

- Frames
- No Frames

- All Classes
